# Measure, Then Show: Grasping Human Evolution Through an Inquiry-Based, Data-driven Hominin Skulls Lab

**DOI:** 10.1371/journal.pone.0160054

**Published:** 2016-08-11

**Authors:** Chris N. Bayer, Michael Luberda

**Affiliations:** AncientAncestors, New Orleans, LA, United States of America; University of Illinois at Urbana-Champaign, UNITED STATES

## Abstract

Incomprehension and denial of the theory of evolution among high school students has been observed to also occur when teachers are not equipped to deliver a compelling case also for human evolution based on fossil evidence. This paper assesses the outcomes of a novel inquiry-based paleoanthropology lab teaching human evolution to high-school students. The inquiry-based *Be a Paleoanthropologist for a Day* lab placed a dozen hominin skulls into the hands of high-school students. Upon measuring three variables of human evolution, students explain what they have observed and discuss findings. In the 2013/14 school year, 11 biology classes in 7 schools in the Greater New Orleans area participated in this lab. The interviewed teacher cohort unanimously agreed that the lab featuring hominin skull replicas and stimulating student inquiry was a pedagogically excellent method of delivering the subject of human evolution. First, the lab’s learning path of transforming facts to data, information to knowledge, and knowledge to acceptance empowered students to themselves execute part of the science that underpins our understanding of deep time hominin evolution. Second, although challenging, the hands-on format of the lab was accessible to high-school students, most of whom were readily able to engage the lab’s scientific process. Third, the lab’s exciting and compelling pedagogy unlocked higher order thinking skills, effectively activating the cognitive, psychomotor and affected learning domains as defined in Bloom’s taxonomy. Lastly, the lab afforded students a formative experience with a high degree of retention and epistemic depth. Further study is warranted to gauge the degree of these effects.

## Background

### A. Sub-optimal instruction on evolution

Although evolution is widely understood to be *the* critical organizing principle in biology as it explains the very development of life on earth [1–3], science advocates have lamented its sub-optimal instruction at the high-school level. In a national survey of high school science teachers, Pennsylvania State University professors Berkman and Plutzer [4] found that only 28% of biology teachers adequately taught the evidence for evolution and presented it as the single most important organizing principle in biology, consistent with National Research Council (NRC) recommendations [5]. At the other extreme, one in eight educators (13%) taught creationism/intelligent design as a valid alternative to evolutionary biology and spent at least an hour of class time presenting it in a positive light. The “cautious 60 percent,” a figure unchanged from that of a 2008 survey by the same researchers, spans the divide between these two poles, endorsing neither evolution nor its non-scientific “alternatives.” Similar findings are reported by the *Factors Influencing College Science Success* (FICSS) survey which targeted 8,310 undergraduate students enrolled in introductory science courses at 55 U.S. universities or colleges across the US in 2002 and 2003. The investigators found that 5.2% of students recalled biological evolution having been taught as a central theme in their high school biology courses, and 17.5% reported that evolutionary biology was not a subject taught by their high school biology teacher [6].

A teacher’s religious beliefs, acceptance of evolution, his/her training, or the lack of confidence to defend it are not the only reasons why evolutionary biology is not, or insufficiently, taught. A personal aversion to–or unwillingness to court–controversy, external pressure from school officials and/or parents, unfamiliarity with laws concerning the treatment of religion in schools, and lack of time are further determinants [7–9].

As summarized by Dr. Eric Plutzer, “students are being cheated out of a rich science education” [10]. This inadequacy results in many forgone opportunities. The National Research Council declared that a deeper understanding of biological systems “both allows the development of biology-based solutions for societal problems and also feeds back to enrich the individual scientific disciplines that contributed to the new insights” [11]. Thus, the degree to which evolution is understood has far-reaching consequences for society’s prospects.

Effective interventions, as recommended by Berkman and Plutzer, would include (1) improving the instruction in evolution that students receive as undergraduates, (2) the requirement for all pre-service biology teachers to take an evolution course, (3) the provision of relevant instructional materials, as well as (4) outreach efforts such as webinars, guest speakers, and refresher courses targeting (pre-service) teachers and biology/science education professors alike.

This paper concerns Berkman and Plutzer’s third recommendation, addressing the provision of improved secondary school science instruction, and in particular the lack of quality inquiry-based materials for teaching human evolution. Although the selection of biology curricula is certainly subject to state- and school board-mandated parameters, teachers preside over varying degrees of discretionary leeway in planning and delivering their lessons’ contents. Charged with imbuing science, knowledge and formative skills to their students, yet constrained by school budgets and mandatory student examinations, teachers face the challenge of identifying and delivering truly instrumental material. Furthermore, as the burden of proof rests on the biology teacher to build a compelling case for a student to validate the theory of evolution, the availability and access to relevant quality labs is essential.

### B. Paleoanthropological hominin evolution labs

Developed with ESEA Title IV-C funding and published in 1995, the *Stones and Bones*: *A Laboratory Approach to Physical Anthropology* program consisted of 20 lessons that explored numerous anthropological topics, offering students an opportunity to study the story of humankind [12]. Investigative-oriented "hands-on" labs also featured replica casts of fossil hominids and focused on “topics including primate behavior and distribution, interpreting archaeological records, primate locomotion and morphology.” These resources are however no longer publicly available, and the current state of knowledge will have supplanted much of the two-decades old material.

Publicly available labs offering instructional tools to high school teachers notably include the “Hominid Cranium Comparison” (The ‘Skulls’ Lab) by Martin Nickels of the *Evolution & the Nature of Science Institutes* (ENSI) of Indiana University [13]. In this lab, students “describe, measure and compare cranial casts from contemporary apes, modern humans and fossil ‘hominins.’” While this lab does include an inquiry-based approach featuring a comparative analysis of skulls, one drawback is that it does not stipulate exactly which specific hominins are to be used, limiting its potential to include discussions of functional anatomy. As it is largely observation-based and not measurement-based, another notable weakness is that it does not hone in on three telltale milestones of human evolution that are readily measurable: the opisthion index, prognathism, and cranial capacity (three variables that are further discussed in section *F*. *Description of lab components and delivery*). This, in turn, might restrict a student from making inferences about the most salient developments in human evolution.

Larry Flammer, also of ENSI, presents the “Chronology Lab”, an adjunct exercise to “The Skulls Lab,” in which students first plot several hominin species’ dates of existence on a timeline chart and then connect them to produce a phylogenetic tree [14]. While a valid exercise, a more complete picture of hominin evolution has emerged since 2001 when the lab was developed, and the lab does not include mention of modern *Homo sapiens* or of *Sahelanthropus tchadensis* on the far ends of the cladogram.

Professor John Roberson of Westminster College, in an article entitled “Investigating Human Evolution Using Digital Imaging & Craniometry”, discusses 6 possible measurements with skulls: (1) braincase size, (2) condylar position index, (3) supraorbital height index, (4) maxillary prognathism index, (5) angle of forehead, and (6), the post-orbital constriction index [15]. He suggests using an array of hominid skulls, including *Gorilla gorilla* and *Pan troglodytes* skulls, and having students take pictures in order to later conduct the aforementioned measurements digitally. Although it is not the purpose of the article, Roberson does not discuss how these measurements might tell the overall story of human evolution.

More recently, Rebecca Price developed an engaging learning module in which students apply an inquiry learning method to investigate “the most common misunderstandings about evolution that humans evolved from chimpanzees” [16]. By guiding students through the 5e inquiry cycle–**e**ngage with, **e**xplore, **e**xplain, **e**laborate, and **e**valuate data–students are guided through the scientific process “collecting data from the casts of skulls of fetal, infant, juvenile, and adult chimpanzees, as well as adult skulls of *Homo sapiens*, *Homo erectus*, *Australopithecus afarensis*, and *Ardipithecus ramidus*.” The exercise is brought full circle when students “ultimately reject the first hypothesis and formulate an alternative: that the ancestor shared human and chimpanzee characteristics.” This valid pedagogical approach thus seeks to demonstrate how scientific conclusions are formulated, with a core learning objective being that physical developmental changes “are a source of heritable variation on which evolutionary processes can act.” Their approach however stands in contrast with our lab in which students analyze a more comprehensive set of fossil characteristics and are guided to draw informed conclusions on particular milestones in human evolution.

ExploreLearning’s *Human Evolution–Skull Analysis* Gizmo™ features an online tool with which the learner can measure three variables (opisthion index, cranial capacity, and maxillary angle) on abstractions of hominid skulls [17]. One notable limitation to this approach is that the learner is not placed in the position of a paleoanthropologist who physically examines the actual skull replicas, and without the context of skulls, students are not confronted with the innate authenticity of the skulls. The tactile element of examining physical skulls is missing. Another limitation is that sketched abstractions are used instead of photographs or 3D models. The two-dimensional analysis restricts the learner in real measurement taking, instead prompting the student to measure cranial capacity by marking the area of the cranium (cm^2^), and then to simply multiply this number by 5 to arrive at the estimated cranial capacity (cm^3^). In addition, as the Gizmo does not educate the learner about dating methods and the chronology of the skull’s appearance, the potential for the learner to appreciate the chronology of the featured variables measured is obscured.

Unbeknownst to this paper’s authors throughout our lab development phase in 2013, Yerky and Wilczynski devised a lab featuring nine hominin skulls in which students identify specialized features and take measurements that enable them to assess the relatedness of the species [18]. The exercises culminate in the placement of each specimen on a phylogenetic tree that also reveals the geological time frame in which each species lived. Variables which the students assess are the foramen magnum index (FMI), cranial capacity, sagittal crest, brow ridge, forehead length, prognathism/snout. For the student’s initial investigation, the authors deliberately chose not to have students undertake instrument-based measurement, e.g. using calipers and protractors, but opted for a literal hands-on approach in which students use their fists and fingers to evaluate the skulls. By circumventing instrument-based measurements, Yerky and Wilczynski’s sought to avoid a concerted focus on measurement and its precision, and to allow students to get a visceral feel for the three-dimensional objects in their palms. Observations on hominins are compared with non-hominin species such as canid and macaque to illustrate key morphological differences. Yerky and Wilczynski thus invite students to have a first look and feel for the fossil evidence, and apply the scientific methods also employed by paleoanthropologists in order to derive “evidence-based conclusions about hominin evolution” widely held by the scientific community. Yet the absence of measurement-based investigation is what differentiates the Yerky-Wilczynski lab from our lab scrutinized in this paper.

### C. Effects of human evolutionary biology instruction on students

The manner in which high-school students are affected by human evolutionary biology education in America is a subject that has received notable attention in the social sciences. Schrein empirically investigates the relationship between the exposure of students to human evolutionary biology (HEB) in high school and their subsequent STEM interest, enrollment and engagement at the university level, drawing on a sample of Arizona State University undergraduate students [19]. She finds that high school science class enrollment was correlated with HEB pursuits in college, and uncovers a significant relationship (r = .134, p = .036) between HEB exposure and students’ interest in a STEM degree. Yet exposure to HEB was found not to be significantly associated with acceptance of human evolution. This finding is reflective of that of Sinatra and Pintrich, who found no relationship between understanding and acceptance as measured by the Understanding Biological Change (UBC) instrument [20]. Nadelson and Southerland however did find a significant relationship between understanding and acceptance of macroevolution (r = 0.47) as measured by the Measure of Understanding of Macroevolution (MUM) [21]. Also Jaksetic found that students who were able to correctly respond to the question linking an understanding of mutations with natural selection were slightly more likely to be more accepting of evolution [22]. In sum, the verdict is still out regarding the extent to which comprehension of evolution begets acceptance of the theory.

Within this context, this study presents data that assesses the degree to which an inquiry-based hominin skull lab enhances student’s grasp of the subject and changes their acceptance of human evolution, to our knowledge a subject previously unaddressed by research.

## Methods

### Ethics statement

This research project was reviewed by Tulane University’s Institutional Review Board (IRB): the Tulane University Human Research Protection Program. Tulane’s IRB determined this study as exempt from a full ethics review on the basis that the research was to be conducted in established or commonly accepted educational settings, involving normal educational practices, i.e. research on the effectiveness of, or the comparison among, instructional techniques and curricula (according to 45 CFR 46.101(b)(1) Research Conducted in Educational Settings: Research). In addition, the investigator had completed the CITI Training Program on Conflict of Interest. Written informed consent was obtained by the interviewed high school teachers–all professionals in their field.

### Research design

This case study relies on key informant interviews held with each teacher–seven in total–who had previously volunteered to participate in the *Be a Paleoanthropologist for a Day* hominin evolution lab that was offered in New Orleans in the 2013/14 school year. The teacher’s perspectives were sought to assess the human evolution lab’s learning outcomes for the participating students.

### Sampling and respondents

As school participation was voluntary and largely contingent on the biology teacher’s interest and cooperation, opportunity sampling, commonly applied in pilot testing scenarios, was used. Yet the sample obtained comprised diverse schools types and geographic locations; from charter schools to traditional public schools, including a Catholic school and a summer preparatory program. In total, seven (7) high schools and one summer program located within the greater New Orleans area participated as 2013/14 pilot schools in this study. Data used in this study were gathered through a project evaluation that accompanied the lab. The authorization to collect these data was granted by each school. Also, the individual depicted in the “striking image” of this manuscript has provided written informed consent for the publication of this picture.

The biology classes in which this “intervention” took place ranged from regular biology classes to honors and AP biology. The biology teachers interviewed, with a range from 2 to 16 years, had a combined 35 years of teaching experience. The teachers’ academic background varied: three had a bachelor’s degree, three a master’s degree, and one a PhD.

### Research questions

This study sought to empirically investigate three research questions:

1. How did the lab accomplish learning?
2. What were the main learning outcomes achieved by the lab?
3. How did the lab differentiate itself from prior (textbook-based) treatment of the subject?

By exploring these questions from the vantage point of the teachers, who were present during the lab, the teacher’s perspective was akin to that of a key informant.

### Sampling procedures and survey instruments

Structured interviews were conducted with the biology teachers serving as key informants (*Appendix 1*: *Structured Teacher Survey Instrument*). These interviews were held toward the end of the Spring 2014 semester, such that the teachers would have had the time to reflect upon the lab and place it in the appropriate context of the course.

### Data analysis

The qualitative data of the teachers were inductively analyzed, substantive and theoretical codes devised, and items tallied. Attention was also paid to the classroom context, noting the type of school from which the findings emerged. An emic focus was applied in order to fully capture the experience and the viewpoint of the key informants (teachers). Coding themes focused on the teachers’ perspective on lab-driven epistemic processes and states, bias and other impediments to student learning, principal learning outcomes, differences of lab vs. lab-free biology curricula, textbook vs. lab treatment of the subject of human evolution, and practitioner vs. teacher considerations. Triangulating the data between the seven data sources allowed us to investigate the lab’s qualities from the perspective of the teachers as key informants.

The investigation also drew on the Data–Information–Knowledge–Wisdom (DIKW) framework to explain the epistemic quality of the lab. The DIKW model, while not universally regarded as a universal theoretical framework or much less a blueprint to derive replicable knowledge (as argued e.g. by Fricke [23]), is nonetheless a useful model to order aspects of the student’s lab experience in terms of data, information, and knowledge acquisition [24–26]. As this paper is concerned with instruction on human evolution as practiced in high-school academics, we however insert “facts” at the beginning and replace “wisdom” with the related “acceptance” of human evolution. We thus adapt the DIKW model to a facts–data–information–knowledge–acceptance schema. The teacher’s verbatim was analyzed through this prism.

### Description of lab components and delivery

While mindful of antecedent approaches, the *Be a Paleoanthropologist for a Day* lab’s pedagogical approach, content, and refinement was designed *ex novo* by this paper’s authors (the instructor curriculum of this lab is downloadable free of charge at www.ancientancestors.org). At its core lies inquiry-based learning (IBL), a component of constructivist learning theory which argues that learning is most profound when a student is, to the greatest extent possible, enabled to autonomously discover knowledge and create meaning [27, 28]. Inquiry-based learning is notably embraced by the National Science Education Standards (NSES) of 1996, highlighting that this technique enables students to “take control of their learning” [29]. “Tell me and I will forget; show me and I may remember; involve me and I will understand,” as stated Confucius circa 2,500 years ago. In their classification of IBL types, Banchi and Bell preset a four-level continuum–confirmation, structured, guided, and open inquiry–of which the second most closely resembles the lab’s particular IBL approach [30]. In *structured inquiry* students investigate teacher-presented questions “through a prescribed procedure”, where however the expected answer(s) is/are not provided. Not instantly providing the student with the answers necessitates the generation of her/his own hypotheses, which s/he substantiates or refutes using the collected data.

Not only is the lab reflective of the maxim “Show, don’t tell!,” it embodies a “Measure, then show!” approach. Students are implicitly told: “Imagine you are the Paleoanthropologist and you found these skulls. Now you are in charge of making sense of them. What do *you* say?” With the hominin skulls in their hands, students are tasked with collecting primary data and guided through observation and measurement. Enabling them to undertake the data-information-knowledge process offers students a method for attaining provable and replicable knowledge and allows them to come to their own conclusions. This exercise, implemented in a period of two to three classroom hours, places the student in the driver’s seat of scientific discovery and knowledge.

Having incredibly survived for millions of years until they were found, fossilized hominin skulls provide irrefutable evidence of human evolution. As such, they not only present a prime vehicle to illustrate evolution, but in-and-of-themselves comprise knowledge objectives. Eleven hominin skulls constitute the lab’s cornerstones, selected on the basis that the skull representing its own specie be relatively intact (they comprised: *Sahelanthropus tchadensis*, *Ardipithecus ramidus*, *Australopithecus aethiopicus*, *Australopithecus afarensis*, *Australopithecus africanus*, *Australopithecus boisei*, *Homo habilis*, *Homo erectus*, *Homo neanderthalensis*, *Homo heidelbergensis*, *and Homo sapiens*).

After students are provided with a lab packet consisting of guided notes, data sheet, measurement instructions, and a glossary of terms, they are given a brief introduction to the field and subject of paleoanthropology, including the provenance of the skulls and who discovered them. Although the lab’s point of departure and primary thrust is paleoanthropology, this mini introduction interweaves findings derived from paleontology, archaeology, genetics and biology to reinforce the lab’s central tenets. Thereafter, the lab’s 11 skulls are introduced and distributed to each lab group. A guided demo instructs the students how to measure three milestones of evolution: bipedality (determined by the position of the foramen magnum), prognathism/orthognathism (determined by the slope of the maxilla), and encephalization (determined by the shape and volume of the neurocranium).

1. The *opisthion index* is a major indicator of bipedality, and the students must calculate it from two different measurements–the full length of skull and the distance of the foramen magnum to the rear of the skull. This index reveals the positioning of the meeting point of the spine and the cranium, where a larger number indicates a more centrally positioned spine, such as an upright human [31].
2. The *maxillary angle* requires no additional calculations, but it does demand the student to connect the data to qualitative features. Prognathism, or the angle of the face, can be measured from the angle of the upper jaw relative to the nose and cheekbones. A more acute angle indicated more protrusion, and this was also associated with other important features such as large teeth and robust lower jaws, implying certain dietary adaptations [32].
3. *The cranial capacity* is measured through a predictive volume measure (due to the fact that many of the skull fossils/replicas do not feature a cranial endocast). Students are asked to measure the cube around the cranium using calipers and then transform that data to the volume of the sphere within the box. Students can then understand statistically what their eyes can see, an expansion of cranial capacity, primarily within the genus *Homo* [33].

The students utilize long jaw calipers and customized bevel angle gauge protractors (tailored to each skull) and apply the measuring methods to the variety of skull shapes. Throughout this time, the students record their figures in the data sheet. Following the facilitated measurement taking, the students conduct calculations and graphically analyze the data. Upon calling out their data in plenary, the student data are compared with that of the instructor’s, which is graphically displayed in the instructor’s presentation. A comparative analysis of each skull follows. Then, the discussion section begins where students are posed higher level questions concerning each milestone and its evolutionary adaptation. What are the advantages of being bipedal? How would the diet differ between prognathic *Australopithecines* and flat-faced *Homo*? What are the benefits of a large brain? An accompanying note sheet stating these lead questions guided the students in their note taking.

Importantly, the ages of the skulls were only revealed after the skull measurement exercise and functional anatomy discussions had taken place and an introduction to material dating techniques had been delivered. Then, with the phylogenetic tree exercise comprising the final synthesis, the skull’s order within the last six million years comes into focus. This concluding activity is a hands-on discussion format where students attempt to physically arrange the skulls, from oldest to youngest, in the order of their phylogenetic tree, enabling students to visualize how the species relate to each other. First students must deduce the oldest species based on their similarity with a shared common ancestor. Then focusing on the genus *Australopithecus*, the students observe the trend from gracile to robust. Ordering the emergence of the genus *Homo* is most important, as it allows students to see the divergence from the large jaw, small brain trend. This is a critical learning moment when students perceive the confluence of many hominin species that coexisted in Africa around two million years ago. The conclusion is the placement of the rest of the genus *Homo*, wherein the expansion of cranial capacity is clearly apparent.

The core strength of the lab’s positivistic approach is its employment of tangible empiricism, i.e. its demonstration of the data-to-theory process. Student’s subsequent argumentation is premised upon empirical data and their systematic observation of the uncovered facts. The facilitator, apart from moderating the interpretation of the data, also engages the students to consider the function of the various physical traits observed. As such, this approach effectively mitigates against the act or perception of a dogmatic “proselytization” of the theory of evolution. Given that the lab was designed to be stand-alone, it does not presume nor require perquisite knowledge of the theory of evolution. Some teachers chose to begin their evolution unit with this lab, and others chose to use it as a building block.

By offering bare-bone paleoanthropology, stripping down the complexities and intrigues of the field into a comprehensible lesson that relays its most salient, non-contentious findings, the material is made accessible and comprehensible to the high school learner. And herein lies the lab’s novelty. While this curriculum is not singular, and like activities are undertaken in many introductory undergrad biology and anthropology labs in the U.S., the unique aspect is that this lab is designed such that it enables scientific instruction of human evolution at the secondary level.

Bloom’s taxonomy–a classification of the possible learning objectives within education–is furthermore a useful framework to explain how the various qualities of the lab engage students. Within the cognitive learning domain (of which there are three; the two others being psychomotor and affective), Bloom’s taxonomy features six hierarchical levels. They are, from the lowest to highest order: knowledge, comprehension, application, analysis, synthesis, and evaluation [34]. Table 1 below describes how the lab exercised all three learning domains and all six hierarchical levels. The lab’s immersive quality and employment of these various learning modes explains how the lab penetrates a student’s consciousness. In doing so, this lab advances a major goal of secondary education–to challenge students to engage the higher-order cognitive domains.

**Table 1:** Lab’s learning domains, modes and elements (based on Bloom’s taxonomy).

*Learning domains*	*Pertinent learning modes*	*Lab-specific applications*
cognitive	apply, analyze, understand, evaluate, remember	• comprehension of instruction • synthesis of data • generation and corroboration of findings • raising new questions
psychomotor	perceive, initiate, adapt, fine tune	• tactile inspection of skulls • learning how to use measurement implements • honing measurement of skulls
affective	receive, respond, value, characterize, organize	• communication such as instructor-student and peer discussion • satisfaction derived from empirical understanding • revision of personal schema/worldview

Currently, this lab is being rolled out in Texas by Robert Dennison under the banner of Rice University’s *Advanced Topics Academy for AP Biology Teachers*.

### Study limitations

The main limitation of the study is the relatively small sample size of 7 key informants–the teachers of the participating biology classes. Thus, the data in the categories derived through the qualitative analysis may not have achieved full theoretical saturation.

Additionally, since the schools participating in the lab did so on a voluntary basis, which was largely contingent on the interest and cooperation of the individual biology teachers, they could be generally counted as progressive schools in New Orleans. That said, since the gamut of school types (private/charter/parochial, public) in greater New Orleans, and furthermore all biology class types (Biology I, Biology II, Honors Biology II, AP Biology) were represented, the effects of sampling bias would be minimal. In any event, this study does not purport to be representative of the general student body of New Orleans, and given the study design, any observed outcomes cannot be construed as necessarily representative or generalizable.

## Results and Discussion

### A. Lab-stimulated data, information, knowledge, and acceptance progression

#### 1. From facts to data

*Facts* are “things that exist, events that occurred, or contingencies that may occur” and *data* are defined as “given factual representations of reality” [35]. Without getting into a semantic discussion, as “facts are considered to be true while people are fallible”, but yet the “reading” of data depends on human mediation, “data” are be considered “human-developed factual subsets of primary information” [36].

In the case of this hominin skull lab, the set of physical, tangible skulls constituted the facts, and students drew on skulls as the source of primary data. One teacher remarked:

“One of the most invaluable things is having that complete set, not obviously entirely complete, but from a geological standpoint, [the lab] covered a really good piece of ground there, and being able to see that progression laid out in front of you in a spectrum is really powerful. It’s not three, it’s not seven, it’s like 13 [skull replicas] that really cover the diversity of hominid species that the students can look at. Being able to take their own data, and trust that someone else that they know collected similar datapoints on other skulls was probably part of that.”

The inherent quality of the material raw facts, in combination with the act of observation and measurement was an experience for each student that constituted a “primary informing.” While not all student measurements were accurate, and were at times corrected by the lab instructor, each student learned first-hand how to collect–and was made responsible for collecting–quality primary data. In order to obtain *primary factual data*, quality assurance–in this case in the form of lab instructor assisting and correcting the student measurements–is necessary [37]. One teacher explained:

“There used to be a theory that said that the ability to follow direction is the heart of the lab. To match your data with the expected data is the Holy Grail. The understanding now, which I agree with, is that the ability to find the data, see what it says, and then analyze its validity is probably what’s more important.” “People will,” he argued, “gravitate towards that personal experience” as it will “stick with people.” “If there is some way to demonstrate through action how this works, it typically is learned better.”

Praising the lab for giving students a taste of real science, one educator remarked:

“It takes a long time to go through the measurement phase. What a lot of teachers do, especially in settings where you are crunched for time, is you give them the data. But the actual measuring helped them appreciate how detail-oriented science can be, and maybe even how mundane sometimes it can be if you have to do this a thousand times, or replicate after replicate. It allowed them to appreciate a little bit what real science is, because they don’t always get that. They think science is memorizing processes and terms and vocabs, and not actually doing the process. They got to do the process this time.”

Even from the vantage point of one of the more well-funded schools, one teacher commented:

“It’s hard to find data that are going to support [evolution], that you can actually measure and look at, and you’re providing that–so that’s a really invaluable service, because it’s a topic around which people will have a difficult time running some sort of inquiry-based lesson.”

Furthermore, the measurement modules positively animated the students: “It was a really great lab. I remember they all had a lot of fun, they all got better at taking the measurements over time.” Similar impressions were relayed by another teacher: “It was a wonderful experience for them, and it’s an excellent way to motivate them.” Thus, in a subject that is taught abstractly if at all, collecting primary data on each hominin’s adaptations fully engages a student’s mind and reinforces the science behind evolutionary biology.

Lastly, interacting face-to-face with the set of skulls, students also recognized an inherent existential quality in the skulls. Students would realize, according to one teacher: “‘It must be real–I’m looking at these skulls!’”

#### 2. From data to information

The lab furthermore charged students to produce information from the data. *Information* is commonly understood “as data that are endowed with meaning and purpose" [38]. As this lab prompts students to themselves observe, measure, describe, calculate, discuss, and analyze, e.g. by comparing and contrasting data points, by answering the stated questions or generating new questions, or by explaining their answers, they were charged with generating information. Prompting their thought processes were the guiding questions put to each student and the plenary discussions throughout the lab. Highlighting these skills that the students hone, one teacher commented:

“I really liked that they are measuring in [the lab] and doing some analysis. It’s important to look at data and draw conclusions from it, and that’s a tough skill for a lot of students. So to give them one more opportunity to do that is always a good thing.”

Deriving information by making sense of the data and furnishing informed answers, was a real challenge for students as one teacher recalled:

“Interestingly enough, the students began to really believe that learning for real is hard. They had to put all this thought into it, graph it, and work. They were like: ‘Man, I thought this was supposed to be fun.’ ‘Well, you did have fun, I’m glad you had fun, and also this is a school. You learn things from the experience.’”

Combining the elements in order to tell a coherent story was the next cognitive process potentiated by the lab.

#### 3. From information to knowledge

Knowledge is commonly regarded as being a “fluid mix of framed experience, values, contextual information, expert insight and grounded intuition that provides an environment and framework for evaluating and incorporating new experiences and information.” Originating and applied in the mind, it requires the knower to organize information into a coherent framework or system. Knowledge, according to Blackler, must furthermore be situated and contested to determine whether it is factual [39]. Knowledge generation, in the context of this lab, involved engaging in a dialectical process with the lab instructor and ordering and synthesizing the information into sensible theses.

Teachers indeed reported that the lab had anchored many of the exposed students. Particular learning objectives met by the lab, according to the teachers, included basic concepts of evolution such as adaptive speciation, descent with modification, common ancestry, and survival of the fittest (see B. Teacher-reported learning outcomes section below). In sum, it allowed students to achieve, as one teacher put it, “a better, deeper understanding of the topic.”

#### i. Referencing the skulls and lab’s findings

To gauge the extent to which the lab had been a formative experience for the students, teachers were asked whether in later discussions the students had referenced the skulls as tangible facts. All seven teachers attested their students had done so, albeit to varying degrees. “People were pretty impressed by the skulls, and definitely referenced some of the different morphological features of them and some similarities and differences when we talked about it later” commented one teacher. Another noted: “While it’s a very specific piece of evolution as a topic, it was something that we could refer back to after we had already done the lab. Just in terms of science in general, it was an experience they could remember.”

The degree of knowledge acquisition is also reflected in one teacher’s observation that in his class several students had invoked skull-specific examples in the context of other scientific subjects discussed in the class. Another teacher remarked that the lab “gave them empirical numbers that they could work with. I definitely noticed that some of my students were able to go back to one specific piece of data that for some reason resonated with them and could use that as evidence for evolution.” Just how vividly some students would recall learning objectives of the lab, and to what lab element she attributes this outcome, is illustrated by this teacher’s testimony:

“Originally they had an assignment where they had to summarize every lab and activity for the whole year. They had to tell us what the purpose was, what the procedure was, what our methods were, and what our final outcomes and conclusions were. They were not supposed to use their materials, it was just an in-class assignment. The student who had to summarize this lab was able to remember the three variables measured, facial prognathism, cranial capacity, and opisthion index. They were able to grasp what we did, three months after we did the activity without referencing the materials, which was a factor of the experience of it. It wasn’t just this is data, we are graphing it, it was physically manipulating the skulls.”

One teacher predicted that the lab-imparted knowledge would stick: “I’ve had kids from last semester this semester come up and ask: ‘Oh, are you gonna do the skull thing again?’ So in a semester, or in a year, or in 5 years, this will be something they will remember from biology class.”

#### ii. Differences in learning outcomes of current vs. prior biology classes

There was unanimity among the teachers that the level of understanding on the subject of human evolution was more elevated in their current lab-exposed class vis-à-vis prior classes that did not benefit from the lab. For one, in the lab students generate substantiated findings as they are made responsible for observing, measuring and generating meaning from facts, which presents a radical departure from prior pedagogy that presented facts and theories as established and a smooth, non-controversial path to knowledge. The lab’s evidence-based approach was characterized by one teacher: “From a general nature of science perspective, [the lab] made clear that these explanations and hypotheses that we come up with are not just subjective people’s guesses. We do measurements, these are standardized, we do dating, and its different types of evidence all coming together.” Thus, mimicking the scientific process positively reinforced the confidence students had in the theory of evolution.

“Seeing the skulls and measuring them, hearing where they came from, and what we look at and measure to compare and date them to piece together this timeline, gave students one more example of evidence for evolution and that scientists are not just guessing, that we have empirical evidence that we use to support the claims that we make.”

The lab’s function as offering a cognitive foundation was emphasized by one teacher who explained: “I would say next to non-existent would be the degree to which my students in the past could have talked about human evolution. A lot of my current students would use this as a way of framing their thoughts around it.” Similarly, another teacher noted: “There is more understanding because they are actually able to communicate about human evolution, actually having concrete things to say.” This point was echoed by another teacher, who held that the students were now

“able to articulate the changes. [The lab] gave students a roadmap for discussing the process of evolution, particularly in humans, from Lucy to us, and from a Neanderthal to us. Whereas a past class said: ‘There was us and a caveman, and my mom says there was a monkey, and I don’t understand how the monkey got to us, and what the caveman had to do with it, isn’t the caveman us?’ This gave them a visual clue, something they could hold, to say: ‘Ok, I see how these are different. You’re telling me based on their age they were around at the same time, I get that they are different, I see how they are related.’ It was very helpful.”

Another teacher described the lab’s potential to be linked with other related themes in biology:

“I especially liked all of the connections to other anatomical features: the use of hands and language, the development of the nervous system that sets us apart from other organisms. That really came back into play, at least in my AP class, through most recent discussions of the human nervous system and the evolution of ganglia and all of these different forms of nervous tissue of different organisms. A lot of students saw how this hyper-specialization surrounding the use of tools, and development of language really set certain species apart from others.” One teacher summed up the lab’s effect on students: “There is definitely a deeper and longer-lasting understanding observable with this class. They now understand for life!”

#### 4. From knowledge to acceptance

The literature, as discussed in the paper’s background section (see C. Effects of human evolutionary biology instruction on students), has sought to understand the interplay between knowledge of human evolution and acceptance thereof. Where authors do concur is that knowledge of a theory does not automatically translate into acceptance. Factors of learning and acceptance highlighted by teachers included a student’s appreciation of the skulls as hard evidence, his/her immersing themselves in the experience and following the lab processes, his/her perceived veracity of methods and findings, his/her personalization, internalization and alignment of the new knowledge with his/her existing belief system, and his/her ability to overcome a negative bias.

Teachers associated student acceptance for human evolution with the quality of *evidence* furnished by the lab. The issue wasn’t as much student denial of human evolution, argued one teacher, as much as the failure of educators to supply hard evidence and a persuasive delivery. “A lot of [students] just don’t know”, explained another teacher, and are skeptical due to misconceptions such as humans having descended from modern apes. “They are on the fence or against it because they think there is no evidence for it. So once they are presented with some evidence, they are more likely to believe it.” Another teacher observed that “A couple of students did make this movement towards believing that there is a lot to be investigated here, that people have actually looked at, and it’s not a made-up theory, there’s hard evidence behind it.” Another teacher thought that those unconvinced might have needed more time to contemplate the meaning of the lab’s findings: “Due to deeply embedded cultural ideas, holding the evidence in their hands helped a lot of them. The hands-on experience with fossil casts convinced them, and even those still in disagreement may eventually come around.” Tangible evidence, therefore, plays a big role.

For other teachers it was engaging learners in the whole process, from data collection to synthesis, which persuaded learners: “There was a group of kids in the class who were like: ‘Evolution isn’t real because we weren’t monkeys,’ and then they went through this and they had a much better appreciation: ‘Oh, I see how it went!’” One educator credited the “data-driven method at arriving at the conclusions.”

Furthermore, students first need to agree with the lab’s methods and findings in order to accept that also humans indeed evolved. In other words, accepting the veracity of the process was a prerequisite to accepting the results. One instructor noted that in his class, “There was nobody who walked out and said: ‘This was all made up!’ Everybody was pretty convinced that that was real science. And that was very compelling, particularly in this population. It was very convincing.” More often than not students “agreed” with the conclusion that also *Homo sapiens* are products of evolutionary pressures one teacher noted, and “in most cases it was a fairly strong agreement.” “There were certainly more people who believed it after the experience than before,” reported another. This observation was echoed by another teacher who recounted: “As a result of this unit as a whole with this [lab] as a supporting agent, they were less towards the Adam and Eve camp and more towards the evolution camp.”

Students, also skeptics among them, now saw how evolution “was scientifically plausible.” One teacher pointed to the skeptical “fence-sitters” in the group: “A couple of students, 2 or 3, were very hesitant at first. But the day after we met they came up to me and said: ‘You know, I was on the fence there–but not anymore.’” For these students, the lab’s findings had sunk in.

One teacher, who presided over a class representing the full range of worldview profiles, characterized the lab as “convincing:”

“To what extent was the doubt factor reduced, mitigated, or else completely trounced? It certainly required more than just the skulls, although it was a big part. More labs, and a variety of evidence [would be needed]. [But the lab] was very compelling. At the very least, someone would say to you that they believe in evolution and given the skulls and given this and given that they certainly understand how. Student X would say: ‘A mouse is related to a whale, and I can see now how a fossil would show that.’ I think they could tell you an abundance of evidence that proves that it must be true.”

An additional prerequisite for accepting the reality of human evolution is that students would need to personalize, internalize and align the new knowledge with their existing belief system. If need be, the new information would need to be reconciled with the preceding world view. Here, as well, bias came into play. For example, according to one teacher, some students were not completely convinced to the point of saying: “Ok, it definitely happened!” One teacher noted that among students who remained doubtful: “there were a couple who were still holding on to a bias against those ideas.”

**Fig 1:**
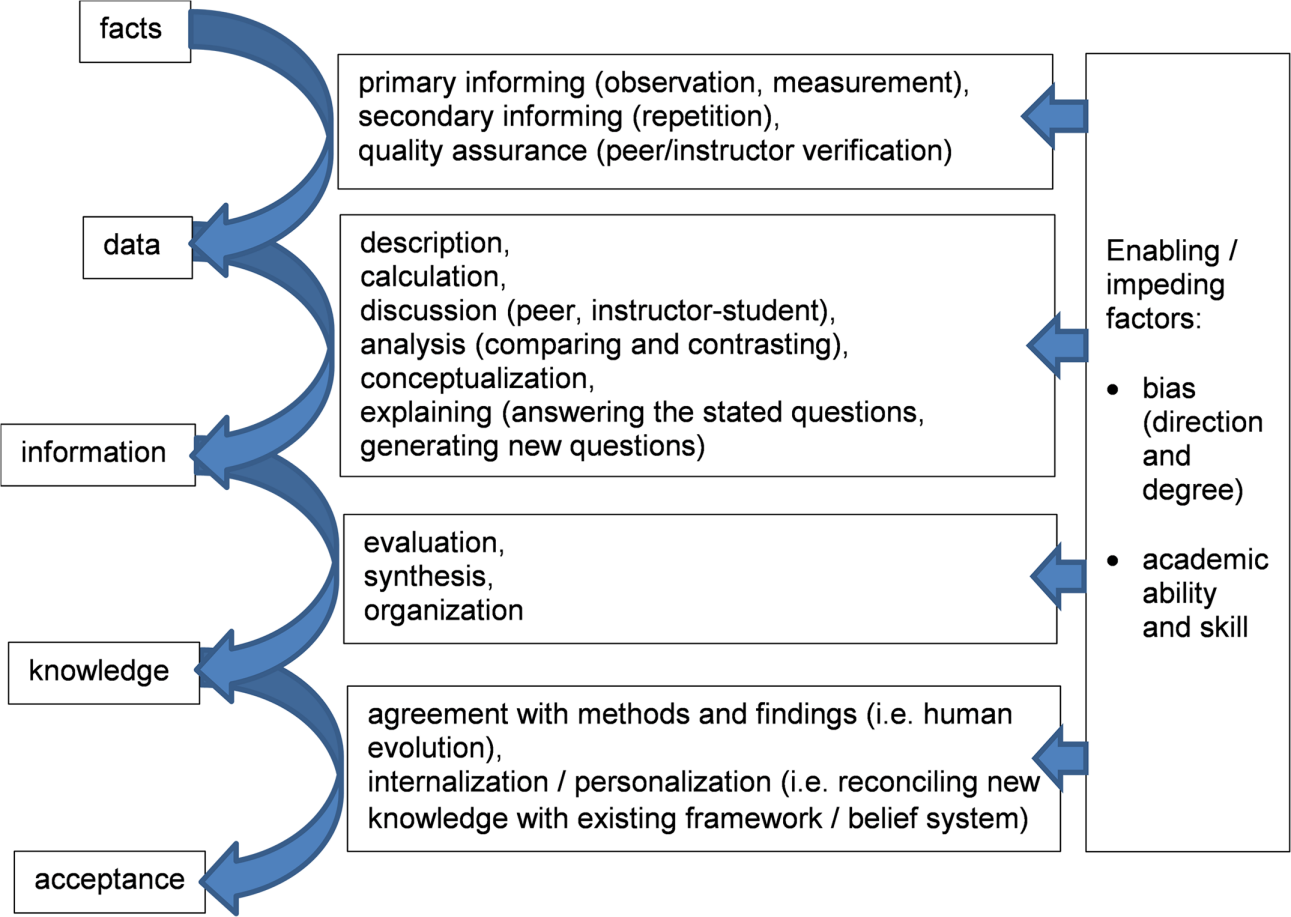
Lab-induced epistemic processes and states.

In summary, the teachers’ first-hand observations describe the nature of the students’ learning progression. Fig 1 below depicts the process students underwent to attain each epistemic level, and also points out limiting factors highlighted by the teachers as is discussed in the next section.

#### 5. Enabling/impeding factors to acceptance of human evolution

Teachers highlighted two principal enabling or impediments to learning about–and acceptance of–human evolution: academic ability/skill and a negative bias.

#### i. Academic ability and skill

One teacher, who referred to Bloom’s taxonomy to explain the challenges he perceived as facing his class, cited the degree of cognitive skill as a notable learning limitation in his classroom:

“Students here at this school, specifically, struggle with anything above application. Analysis, we get there, but it’s a struggle, and synthesis, which is what, really, we’re asking them to do in an advanced Biology class, to synthesize all of these different data points and concepts into these very broad organizing principles, one of which is evolution, is a big struggle for them.”

This however was the only instance an interviewed teacher identified this particular limitation. For the other schools, all three learning domains and six hierarchical levels were exercised according to the interviewed teachers.

#### ii. Bias interference

Bias, which may be defined as a preconception, predisposition or inclination to be for or against something, is often present, to varying degrees on a culturally controversial subject such as human origins. In some classrooms teachers noted little or no bias that would affect a student’s perception of the lab or stand in the way of the lab’s knowledge objectives. One teacher, for example, noted that in his class, “Most of those two classes were pretty open to it and were not coming at it with a whole lot of bias.” In other classrooms teachers observed a bias interference. The importance of cultural and academic background also came into play, as, explained by one educator, “A lot of students find it easier to rely on things they have been taught in their prior experience.”

Naturally, bias can cut either way. A teacher in a private school recounted: “This group of students especially has been very pro-evolution, so I never have had any push-back or any disagreement. They honestly saw it as an opportunity to get more evidence to go argue against people who didn’t understand evolution.” In one New Orleans’ Biology I class, “Nearly all of them agreed with the idea that species are transitioning, with only a few students in disagreement.” Also in a Catholic high school there was no implicit conflict reported between religion and science.^i^ In an honors biology class for example, the teacher reported that her students were “pretty sure that evolution had occurred. Even a lot of them were religious, they said ‘I believe in God, I go to church, but this is where the evidence is.’ If anything it was a confirmation, a cool experience. ‘Now I have more evidence for these beliefs that a lot of people around me do not share.’”

Yet in other classes, students were reportedly “pretty split” on the subject of evolution.

“Kids on the first day of the evolution unit were like: ‘How do Adam and Eve relate to this?’, and I’m thinking it’s a fairy tale, it doesn’t relate to it really at all, it’s a cool story. I had some kids who were very against it, and they are very vocal, and I have kids who say evolution occurred, and they were very vocal.”

In one class exhibiting particularly diverse views, according to the teacher, a quarter of the class had accepted evolution due to prior knowledge, a cohort that he described as “the highest achieving and the best educated students that I received.” Their response to the lab was: “‘Been there, done that, it makes sense.’” The majority of students in that particular class were undecided, and there was reportedly a really small minority of students with preconceived notions opposed to accepting evolution: “I go to church every Sunday and they say [evolution] is baloney.” Just how “sticky” contextual bias can be, is revealed by student comments such as: “‘That can’t be true because my mom said it wasn’t true and that seems ridiculous!’” Yet this teacher found that even the skeptic’s mind could be changed: “They were malleable, much more so than I imagine if you were to go an hour outside of the city.” This teacher’s main strategy for circumventing bias is to shift student’s focus on knowledge objectives.

“The way I message is: ‘You can believe this or not believe this, but this is what you need to know to understand this scientific process that you will be tested on.’ And that actually, I found, it pushes the debate to the side, and we are just talking about the science. Given the urgency around testing, and the push for these students to really show high levels of mastery, they want to know that science, and they want to be able to demonstrate it at a really high level.”

Then,

“Everyone is focused on how do we memorize that, know it, explain it, and tell them the importance of it. So in that way, they are really focused on the science. Which means that for a lot of the kids, when I said: ‘Fossils show evolution,’ they said: ‘Oh yeah, all those skulls.’ ‘When he says proof of evolution, there were the fossils.’ So they are making that connection passively.”

Differing degrees of bias were reported by the educators. One teacher, who perceived her student’s views as rather entrenched, commented: “I think most of them thought the activity was interesting, but it didn’t necessarily change their mind about whether evolution occurred.” One teacher recounted that students in his class showed resistance to the lab’s very methods: “Actually, there were two or three students who were apprehensive about applying the same principles to humans.” In another class, the teacher noted that bias would not necessarily cause his students to ignore or deny facts: “They are not a crowd that vocally opposes evidence as it is given to them.”

**Fig 2:**
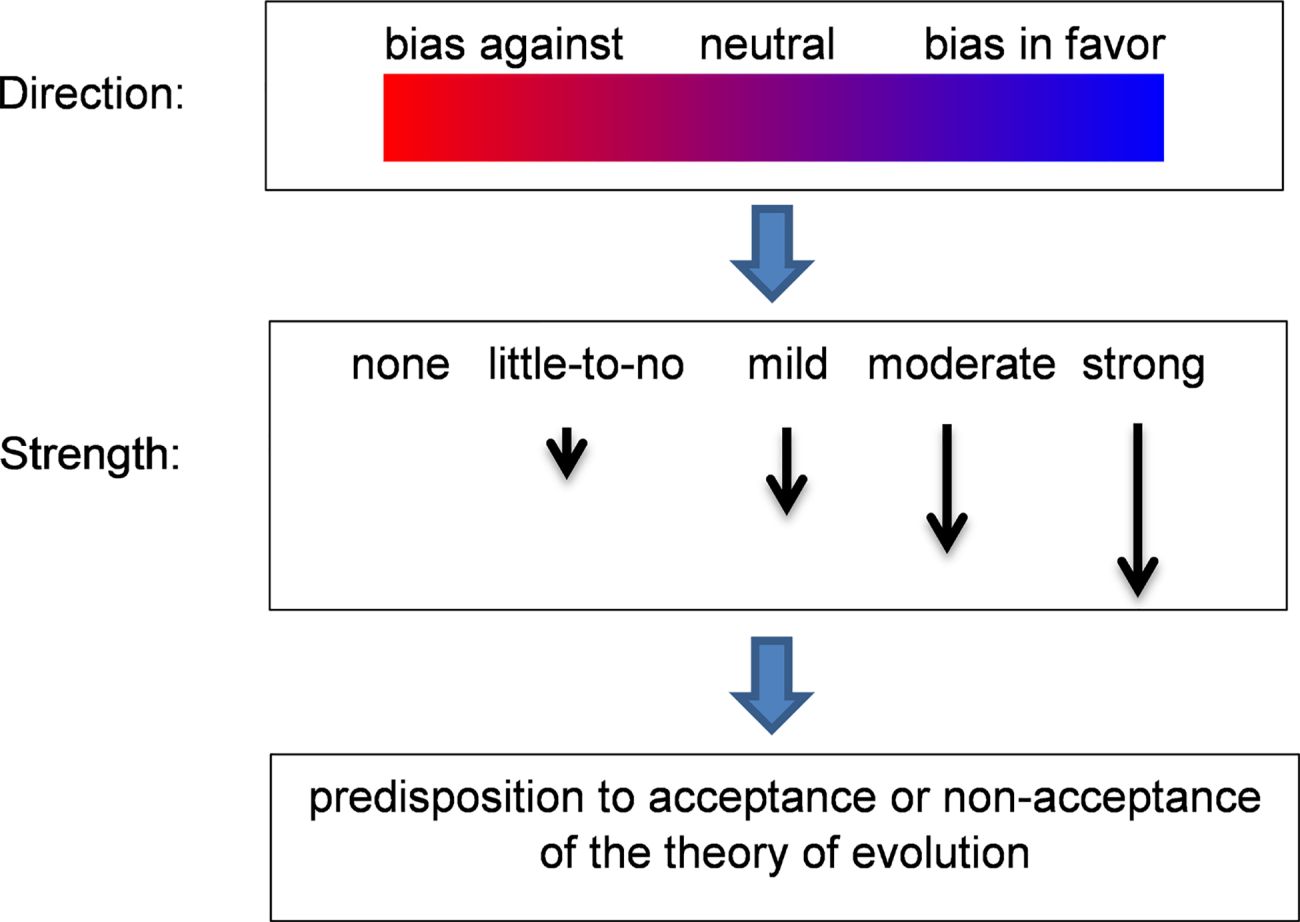
Bias direction and strength.

In sum, the direction and degree of bias reportedly played a role in the student’s acceptance in the lab’s methods and findings, as illustrated in Fig 2 below.

### Teacher-reported learning outcomes

On the whole, according to the teachers, the lab deepened students’ grasp of salient concepts in evolutionary theory. According to them, the lab drove home basic concepts of evolution such as adaptive speciation, descent with modification, common ancestry, and survival of the fittest. Teachers also associated elements within process with student acceptance in the notion of human evolution.

#### 1. Associating fossilized hominin crania with evolution

The inherent empiricism of interacting with fossil hominin crania replicas was noted by each teacher, allowing students to appreciate that human evolution is evident in the fossil record. From the moment the students first enter the classroom on the day of the lab, the skulls at the front of the room stare back at them. “A lot of students are just jazzed to get something that appears as though it came right out of the ground,” remarked one teacher, “and some students take that in a distracted manner ‘Look at this skull I got here.’ That’s going to be common at least for a little while. But just about everyone buckled down.” After the fossils were introduced and first impressions were made, students were in a position to make the connection between fossilized hominin crania and evolution. “The lab itself was not an overarching lesson on evolutionary theory,” commented one teacher, “but instead a specific, yet quite personal, instance of evolution at work. For some students it was their first time seeing their own species’ skull, and the excitement was clear for every new group of students in this study.” Similarly, another teacher noted that the lab “gave them a physical, visual, kinesthetic way of connecting with evolution because it’s such an abstract idea at times. It’s hard to see in front of you like you can see a pH paper turning colors. [This lab] gave them a visual, physical, tactile-manipulation-of-the-skulls experience.”

#### 2. Grasping “adaptive speciation”

One principal theme of the lab is adaptive speciation, or evolutionary branching, a concept explored through anatomical analysis and comparison, i.e. measuring and interpreting three major anatomical adaptations expressed in different degrees across the eleven featured species. Although the skeletal analysis was limited to the skull, the cranial traits paint a clear picture of hominin evolution–from a small brained arboreal species to a large brained bipedal species, with robust *Australopithecus* variations emerging and eventually going extinct. One teacher commented: “They got to see the anatomy of it. So many of the skulls look much like ours, and seeing how it was different and why it was different helps their understanding.” In a similar vein, another teacher commented that “I really liked this discussion of anatomy as a feature that we can use to compare species. You can look at all kinds of forms, and get a pretty good understanding of where they fit in an evolutionary tree.” Indeed, within two classroom periods, this lab sufficiently enables the learners to induce the hominin’s temporal and relative order, yielding, according to one teacher, “a really good understanding of evolution as it actually occurred over time.” Another teacher referenced the lab’s phylogenetic tree exercise, in which, towards the end of the lab, students induce the position of each featured specie based on previously collected data and findings: “My favorite part was putting the skulls together in that phylogenetic tree” she stated.

Anatomical analysis and comparison furthermore clears up one major misconception of evolution: the linear version propagated by popular graphic of man slowly standing upright. “‘So we used to be a monkey or something like that?’” is a question students “always” reportedly asked one teacher. “No, we share ancestors” he then explains. “The lab does a good job showing the branching nature of evolution instead of this one thing becoming another”, he concluded. Another teacher described the same stumbling block, and how the lab helps students overcome this obstacle:

“They got to see real examples of anatomical changes. They have these misconceptions that it went from monkeys to humans, so [the lab] gave them ideas what came in between. That is a huge misconception that there were monkeys and one day one of them stood upright and suddenly we’re humans, and that is something they don’t understand is that things came in between and how they lived and what they looked like. So it really helps them see the anatomical changes and relate them to why the changes came about. What struggles were they facing that made it advantageous to have a larger brain, or a smaller brain, or to have a larger jaw or a smaller jaw?”

These observations were echoed by another teacher: “So instead of just thinking ‘Humans came from monkeys? That’s crazy!’–they were like: ‘Well no, there are these intermediate species, these other things happened, and it’s not really that simple of a story.’” Consequently, this teacher credited the lab with getting “a lot of them to start thinking about it, and thinking about it more critically.” In sum, students began to appreciate that evolution had elicited a far more multifaceted sequence of hominin taxa than hitherto commonly imagined or held.

#### 3. Picturing “descent with modification” and “incremental change”

Instead of first presenting the key theoretical aspects of evolution, and then investigating how this evolutionary theory is played out in this specific context (deductive approach), the inverse (inductive) method was applied. The facts–the skulls–came first. Theory, which revealed itself in the course of the lab, was thus an outgrowth. For example, to behold the great variety and similarities among the prime relics of hominin taxa is a cognitive aid towards accepting the principle of descent with modification. One teacher noted: “Most students certainly saw there were differences in the skulls, and that one skull looks like us. And the further back in time you go, the less the skulls look like us. And in between, there were some that kind of look like us, but kind of didn’t.” The notion sinks in that time renders malleable even bone. Another teacher remarked:

“it answered a key question for a lot of students which is: ‘Why does the monkey at the zoo never become a human?’ In other words, seeing how gradual this was, this took a really long time. And being able to hold something that was really old was helpful in them gaining an appreciation for that. That this is not a fast thing, and that is why a monkey at the zoo does not become a person.”

One teacher predicted that even after the summer holidays his students would have certainly retained that “the skulls were different, that some look like us, some didn’t, and that they certainly change over time.”

Another teacher described the realization that incremental change occurs over generations as a watershed moment among his students, also tying this insight to the bushy model of human evolution:

“The opportunity to first of all see and interact with the trajectory of human evolution was very powerful for many of the students who couldn’t conceptualize incremental change. Being able to see the plethora of skulls was very meaningful and really brought that to life. Secondly, and maybe the most impactful, was that the variety of skulls, in conjunction with the lesson, really made it clear that this was not a single trajectory, and that it was not a quick one at that. For them to see that there were lots of dead ends, that there were lots of simultaneous relatives, gave them a much deeper understanding of evolution.”

Thus, with empirical illustrations in hand, students peered back through the eons, the concept of descent with modification was made accessible, and a more nuanced, complex story came to light.

The lab served as a foundation from which the teacher could later extract further lessons. One teacher for example, who saw the lab as relevant to “our whole evolution unit”, used the lab as a springboard: “The subsequent discussions we had about what they saw, it was clear to them how and why changes came about, why there was modification, and why certain things were passed down and certain things were not.”

#### 4. Comprehending “common ancestry”

Once a student had accepted the possibility, even plausibility, that hominin lineages were not static but changed morphologically across time, the notion of common ancestry no longer required a leap of faith. One teacher thought the lab “did a good job of relating humans to our ancestors and to our closely related primates.” Furthermore, an array of skulls with some shared and some unique features begs the question of how they relate to each-other. Another teacher, who had positioned “common ancestry” as “a big buzzword for the year”, stated: “If you were then to ask them next August, after they have been on summer break, and they’ve really forgotten everything: ‘Did man come from monkey’, people would say ‘No.’ If you said: ‘Did we descend from a common ancestor’, most people would say ‘Yes.’”

The examples of nearly a dozen hominin species also offered a logical point of departure with which to comprehend common ancestry back through time. Admitting that it was beyond the expectations he had of his biology students, one teacher noted:

“Lately I had a couple [of] discussions with students where they were talking about finding it difficult to grasp the idea that life went from being molecules that were inanimate to being these animate molecules that are now spread through many different life forms. At least through [Ancient Ancestors] and through the work that you guys did, I think that most students were at least grasping this idea that human life shares these common ancestors that we interacted with, and there is evidence of that, and it’s not a logical jump to talk about how primates are more related to one another than flowers, and going backwards it creates this jumping off point for getting to this really difficult molecular question: ‘If that did happen, how did it happen, and how might we go about investigating it?’”

Thus, although the lab did not hammer home the idea that evolution is the central theme that ties together all living forms, the implication of a common ancestry far beyond hominins was a new realization for many students.

#### 5. Understanding “survival of the fittest”

The concept of the survival of the fittest, related to the idea of adaptive speciation and integral to a proper understanding of evolution, was furthermore demonstrated through the lab using our own genus. “It wasn’t just realizing how humans were a breed apart that sunk in,” explained one teacher, “the nature-species interplay also came to light with the realization that survival of the best adapted applied not only to distant ancestors but to humans, well even modern humans, opening up their minds to the fact that ‘Oh, humans have adaptations, too!’”

The lab also drove home the point that *Homo sapiens* are not excluded from the interaction between selective pressures and our adaptations. “It was mind-blowing to a lot of students that there were other hominids around at the same time, that they interacted with each-other, and there is evidence of these interactions”, recounted another teacher.

“And their imaginations ran wild with this idea of Neanderthals and humans shivering in the wintertime in caves. It was a really powerful, not just in social terms, but also in artistic and self-evaluative terms. With a lot of the extinction events that are happening now and the climate change that will happen in the future, there’s this circular going back to lots of species have died in the past, lots of species will die in the future, are we going to be one of the ones that die or survive? There’re lots of changes going on in earth’s climate now, there were a lot of changes to earth’s climate back then, how have those things affected the ways organisms have evolved?”

The lab thus effectively turned the index finger squarely at the inquirer: “There was a really cool social aspect to it as well” remarked the same educator. “We’re people studying people, and that falls underneath this branch of biology but it also falls in this branch of social science, and I think there were a lot of students who were really inspired by that mixture, which you don’t always get in your science class.” The implications begged further exploration.

The Darwinian understanding of fitness and survival was subsequently deepened by another teacher who, in consequent classes, pursued these themes: “There’s a misconception about natural selection and the strong people survive and everything else dies out when it’s not necessarily black and white like that. So that’s something I chose to focus on.” In sum, the lab also afforded the students a more nuanced understanding of “fitness”–one premised on adaptation.

### Practitioner vs. teacher delivery of lab

When asked whether they could see themselves also delivering this lab, provided with training and the appropriate resources (skulls and measurement tools), each teacher agreed. The responses ranged from “Oh yeah, I would love to get some of those skulls in schools—I would do that in a heartbeat!” to “I believe that I could.” Yet the teachers mentioned the need for “some sort of basic teacher training” and that “video tutorials would be helpful.”

The possibility of then also tailoring the lab was highlighted by one teacher who said: “I think it’s really cool and I can’t wait to do it with my students and have a set that has the scientific research behind it–the think-tank–but also being able to take it and make it my own, focusing on a theme such as evolutionary fitness or survival of the fittest, or whatever concept I want to focus on.” She went on to note that she would “also be interested to see how it would be different if I did it at the beginning as a welcome introduction and take it from there.”

Nevertheless, the teachers also expressed distinct advantages of having a paleoanthropologist deliver the lab. The “WOW factor” would “quiet kids long enough to listen.” While there was “a certain familiarity and a belief around [the teacher’s] capabilities,” an outside instructor, e.g. someone from Tulane, was not going to be called “ridiculous.” In other words, the guest lecturer would bring an unparalleled “cachet” to the subject matter. In addition, a guest presenter would provide the teacher with academic support for evolution, effectively reinforcing the teacher’s own stance on the veracity of the theory of evolution. “If a student asks: ‘Do you believe in evolution?’, all of a sudden I get put in this proselytizing position when I say: ‘Yes, and this is the only thing.’ Whereas to say: ‘This is a scientist from a research university that you all know and he is here to share some science with you today’–that’s different.” This sentiment was echoed by a second teacher: “Evolution was the central theme in this course, and it was good that it wasn’t just me pushing it.” The appeal for external reinforcement is especially relevant in light of Berkman and Plutzer’s findings revealing a “cautious 60 percent” of equivocating high school biology teachers.

A second argument in favor of having a practitioner deliver the lab is to hear it all from “the horse’s mouth”. As one teacher explained: “I like my students to hear from people who have done actual research so it’s not so abstract to them. [He]’s a regular guy, you can do it. In a year or two you could be doing this.” Another teacher alluded to the new paradigms unlocked: “It was a good lab for them to interact with a type of science that they don’t encounter often. That lab showed the class that it’s pretty cool to travel to these exotic places, having an adventure with it, and that science isn’t just about reading some directions and pouring something into a jar.” And although this study’s data do not show that lab participants had an immediate interest in their own personal pursuit of science “it was important for the students to see careers built around the application of science” emphasized one teacher.

Yet a third argument in favor of having an outside expert deliver the lab was offered by one teacher in regards to the student perception of skull veracity. By having an external presenter bring his own skulls and deliver the lab the “awe effect” was maximized. According to one teacher, “Had I opened up my cabinet and said: “Here these are!” everyone would have said: “Oh, you either bought them at *Toys“R”Us* or they are fake. But having someone from Tulane University come and present this gave them a greater appreciation for it.” This instructor, who faced a particularly biased group of students, further explained: “Having an outsider come in definitely provides a buffer for the ‘doubt cohort’, a reaction that also goes for the skulls themselves. If an expert says: ‘These are the real models of the real deal,’ it pushes kids past the doubt. There is no question that the fact that they came in this big box all packed up, and they came from somewhere else, that made them special and authentic.”

## Conclusion

This study set out to explore how the lab achieves learning outcomes, the nature of those learning outcomes, and how the lab differentiated itself from prior approaches to the subject of human evolution. The teachers reported that most students readily engaged with the lab’s scientific process and readily followed its data-driven approach, validating the lab’s intent of making this particular science accessible at the high-school level. The teachers characterized the lab’s student-driven process of turning facts to data, information to knowledge, and knowledge to acceptance as imparting to their students a formative experience with a degree of epistemic certainty that their previous students had not achieved. They furthermore reported that the lab effectively activated the cognitive, psychomotor and affected learning domains as defined in Bloom’s taxonomy. In sum, the surveyed teacher cohort unanimously agreed that the lab’s hands on, inquiry-based and metric hominin skull analysis was a pedagogically excellent method of delivering knowledge acquisition on human evolution.

Whereas the study of Paleoanthropology is technical, complex and nuanced, thus often preventing substantive access at the high school level, this lab extracts the most salient and uncontroversial concepts of the field and presents them in an accessible, hands-on lab. The lab gives students access to the same hard data available to paleoanthropologists, allowing them to analyze and interpret three hallmarks of hominin evolution–cranial capacity, maxillary prognathism, and bipedality. In this way, the learner is empowered to autonomously form impressions and draw conclusions. A thorough understanding of the process by which human evolutionary history is inferred consequently begets confidence in the conclusions reached. In conclusion, combining empiricism with experiential learning allows the learner to reach new levels of epistemic depth on the subject.

The burden of proof rests on biology teachers to make a persuasive case on behalf of human evolution in the classroom. An inquiry-based introduction to Exhibit A–the set of hominin skulls–is a powerful means to this end, as the findings of this study suggest. Instead of working backwards, presenting the theory and supplying supporting evidence, this lab’s approach works from the bottom up, starting with the fossil skull replicas, allowing the students to construct their own scaffold of knowledge. Combining the empiricism of fossil skull replicas with the inquiry-based learning approach produces a lab that excites, motivates and equips students to unravel salient facts and about human evolution. “Nothing in biology makes sense except in the light of evolution” as Theodore Dobzhansky's succinctly stated [40]. Decades later, educators continue to search for the best ways of telling the story of human evolution to today's biology classes. This study finds that the lab in question effectively brings the subject of human evolution to life, and–as the subject concerns our own distant roots–closer to home.

## Perspectives

These preliminary findings lead us to propose the conduct of a large-n study featuring a case-controlled and longitudinal knowledge, beliefs, attitudes, and behavior impact design. One control group could for example be taught the same material in the conventional textbook form, and another control group would carry out another paleoanthropology lab featuring a different pedagogy (e.g. that of Yerky and Wilczynski). Of interest would be to test the various groups’ actual academic performance as well as gauge their pre- and post-knowledge, attitudes and beliefs vis-à-vis the subject of evolution. How would the “intervention” group’s test performance compare to that of “control” students, i.e. to what degree would the featured lab’s pedagogy produce superior learning outcomes and confidence in the veracity of the theory of evolution?

By allowing high-school students to learn about their distant ancestors and imparting the fundamental findings of paleoanthropology, an eye-opening, paradigm-shifting experience was made possible. As the students soon learned, paleoanthropology is not just for paleoanthropologists, and the skulls are far from dead and silent. If you listen, they will speak.

## Appendix 1: Structured Teacher Survey Instrument

### A. Teacher profile

1. How long have you been teaching?
2. How long have you been teaching biology?
3. What is your educational background?

### B. Assessment of lab

1. Did the *Be a Paleoanthropologist For a Day* lab enhance the students' understanding of human evolution (descent through modification)?
2. If yes, how so?
3. Did the *Be a Paleoanthropologist For a Day* lab deepen the students' understanding of evolution as the primary organizing principle of biology?
4. If yes, how so?
5. In consequent classroom discussions, to what extent did students agree or disagree with the material presented to them by Ancient Ancestors (AA)?
6. Were there any students in the class who fully accepted the notion of human evolution who previously had not?
7. If so how many (percent)?
8. In later discussions did students reference the skulls as tangential facts?
9. In your opinion, what presented themes were insufficiently developed?
10. What themes were presented in too much detail?
11. In your opinion, what presented themes were insufficiently understood?
12. Was there a perceivable difference between the level of understanding of human evolution of the current class versus prior classes that did not benefit from the lab (but treated the subject with a textbook-based approach)?
13. In your estimation, how does the understanding of the material differ between a lab vs. a textbook-based approach?
14. Do you have any other comments regarding the impact that the lab had in the minds of the students?
15. Do you have any other comments?
16. Provided with training and the appropriate resources (skulls and measurement tools), could you see yourself as a teacher also delivering this lab?
